# Modeling Collective Animal Behavior with a Cognitive Perspective: A Methodological Framework

**DOI:** 10.1371/journal.pone.0038588

**Published:** 2012-06-26

**Authors:** Sebastian Weitz, Stéphane Blanco, Richard Fournier, Jacques Gautrais, Christian Jost, Guy Theraulaz

**Affiliations:** 1 Université de Toulouse; UPS, INPT; LAPLACE (Laboratoire Plasma et Conversion d’Energie); Toulouse, France; 2 CNRS; LAPLACE; Toulouse, France; 3 Centre de Recherches sur la Cognition Animale, UMR-CNRS 5169, Université Paul Sabatier, Bât 4R3, Toulouse, France; 4 CNRS, Centre de Recherches sur la Cognition Animale, Toulouse, France; Cajal Institute, Consejo Superior de Investigaciones Científicas, Spain

## Abstract

The last decades have seen an increasing interest in modeling collective animal behavior. Some studies try to reproduce as accurately as possible the collective dynamics and patterns observed in several animal groups with biologically plausible, individual behavioral rules. The objective is then essentially to demonstrate that the observed collective features may be the result of self-organizing processes involving quite simple individual behaviors. Other studies concentrate on the objective of establishing or enriching links between collective behavior researches and cognitive or physiological ones, which then requires that each individual rule be carefully validated. Here we discuss the methodological consequences of this additional requirement. Using the example of corpse clustering in ants, we first illustrate that it may be impossible to discriminate among alternative individual rules by considering only observational data collected at the group level. Six individual behavioral models are described: They are clearly distinct in terms of individual behaviors, they all reproduce satisfactorily the collective dynamics and distribution patterns observed in experiments, and we show theoretically that it is strictly impossible to discriminate two of these models even in the limit of an infinite amount of data whatever the accuracy level. A set of methodological steps are then listed and discussed as practical ways to partially overcome this problem. They involve complementary experimental protocols specifically designed to address the behavioral rules successively, conserving group-level data for the overall model validation. In this context, we highlight the importance of maintaining a sharp distinction between model enunciation, with explicit references to validated biological concepts, and formal translation of these concepts in terms of quantitative state variables and fittable functional dependences. Illustrative examples are provided of the benefits expected during the often long and difficult process of refining a behavioral model, designing adapted experimental protocols and inversing model parameters.

## Introduction

Collective animal behaviors have generated an increasing number of studies over the past decade [1]–[3]. These phenomena can be observed at all living scales, from bacteria colonies [4] to bird flocks [5], fish schools [6], [7], insect societies [8], [9] or herds of gregarious vertebrates [10], [11]. These collective behaviors are typically governed by self-organized processes resulting from many direct or stigmergic interactions between individuals and they generally lead to the formation of dynamical patterns whose temporal and spatial characteristic lengths are much larger than the typical range of individual interactions [12]–[14]. Understanding how interactions between individuals control collective behaviors is a challenging problem for a growing research community in biology and statistical physics [15]–[18]. Indeed this is a required condition to establish a continuous causal link from studies dealing with the neural and cognitive basis of individual behavior and studies dealing with collective behaviors in which these neuronal or cognitive processes are involved (e.g. [19]–[22]).

Addressing these interactions starts with the enunciation of presumed sets of behavioral rules that are inspired by and confronted to experimental observations. These sets of rules that define the suggested behavioral model are most commonly of statistical nature: Individual behavioral mechanisms are characterized by the individual’s probabilities to perform a given action (e.g. changing its own direction of motion in a given time interval) or their probabilities to undergo a transition from a state A to a state B [23]–[25], many of them depending on external stimuli (e.g. information about conspecifics and environment). In methodological terms, a very demanding task is that of maintaining as sharp a distinction as possible between the behavioral model, enunciated as a given combination of biological concepts, and its formal translation into mathematical expressions that may not be unique as it depends on the retained set of quantitative state variables and involves fittable functional dependences for each statistical response to stimuli. Only when this distinction is made can the model be criticized for its biological pertinence and the formal translation for its rigor. In this sense, establishing that the model is compatible with the corpus of knowledge in neurosciences, animal cognition and behavior and with available observational data is one issue; using observational data to fit the functional dependences is quite a different one, although both issues are commonly addressed using the very same statistical techniques as far as observational data are concerned.

Historically, the second issue was left aside in a first approach to the understanding of the mechanisms underlying collective phenomena. The objective was to demonstrate that the diversity and complexity of the behavioral patterns observed in swarms, flocks, schools and crowds may result from relatively simple interactions between the individuals [2], [26], [27]. It was therefore sufficient to build biologically meaningful behavioral models and check, using numerical simulations, that these behaviors led to dynamical patterns qualitatively similar to the addressed ones [10], [28]–[30]. This first step was essential as a justification of the forthcoming long term researches toward more quantitative assessments. It has permitted, in particular, to test alternative hypotheses about the behavioral mechanisms taking part in a given collective behavior. However, one of the essential conclusions was also that very different individual mechanisms may reproduce similar collective phenomena. For instance, [31] and [32] have both proposed models for the clustering of objects by ants moving at random in a two-dimensional space. In both models an unloaded ant encountering an object on its path picks it up with a probability 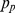. In [31] 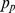 depends on the number of objects encountered during a given preceding time interval (which is a rough indication of the object density in the neighborhood), whereas in [32] unloaded ants pick up every object encountered (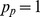). Despite this important difference, [32] highlights that the clustering dynamics of both models are qualitatively the same and close to the experimental observations.

Although this first historical phase is far from being over (numerous qualitative or semi-quantitative studies are still today offering very useful conceptual contributions), an increasing number of recent studies use quantitative approaches in which macroscopic quantities are measured at the group scale in order to characterize the collective dynamics. These measurements have permitted to carefully fit the functional dependences so that the observed collective patterns were quantitatively reproduced [33]–[35]. These more detailed explorations have essentially confirmed the previous conclusions, in particular that with adequate fits, biologically different models at the individual scale are able to reproduce the same collective dynamics, even from a rigorously quantitative point of view.

A very strong consequence of such observations was to establish that it was biologically meaningful to think in terms of renormalization groups, universality classes and asymptotic theory for all studies concerning the dynamics of collective patterns (as was already established for macroscopic physics [36], [37]). This led to numerous research efforts toward the identification of minimum behavioral models associated to some of the most challenging collective behaviors, in particular with respect to fish schools and bird flocks [16], [28], [29]. However, in the context of theoretical biology, these efforts are only relevant to the understanding of the collective behavior itself, closing the door to the above mentioned neural and cognitive direct connections. Researchers dealing with minimum behavioral models do not claim that the considered animals actually follow minimal rules; they state that a given collective pattern dynamics is best described with a behavioral model that may serve as a reference for all analyses at the collective scale. The parameters of these reference models may then be defined as effective parameters that are related to neuronal and cognition details. But how they are related is not an issue : two distinct sets of individual behavioral rules leading to the same effective parameters are indeed the very same model at the collective scale. From this point of view, the fact that we observe it to be impossible to discriminate between them using collective measurements only is a validation of effective parameter approaches and nothing like a practical difficulty.

Researchers equally involved in the understanding of collective dynamics in animals may as well conclude from the same facts that further observational data are required as soon as they are interested in the analysis of individual behaviors at the level of details of neuronal and cognitive processes for a particular species in a given (ecological) context. However, choosing an adequate experimental protocol to decipher between alternative models often proves to be a very difficult task. For instance, a model of the formation of the dominance order in social wasps based on threshold reinforcement has first been experimentally verified from dominance behaviors measured at the individual scale [38], [39]. However, some years later, the same authors questioned the occurrence of this reinforcement mechanism because the empirical data may as well be explained by preexisting differences among individuals [40]. The available experimental behavioral data were therefore not discriminative, even at the individual scale.

All this illustrates that we are still today at the stage of methodological reflections and regularly have to go back to quite basic questions: When attempting to identify components of the individual behavior, what are the respective roles of collective observational data and more specific experimental protocols in terms of model validation and function / parameter estimation? Are there criteria that can guide the design of an experimental protocol? The aim of this paper is to address these questions in the particular case where laboratory experiments can be designed providing observational data that are fully independent of the initial collective observations. This means that we leave aside the more difficult question of designing individual observation protocols based on the very same experiments (or field observations) as those allowing collective quantitative measurements. We start from a published experimental study on object clustering in ants. Six different individual behavioral models are constructed, with rules in terms of statistical responses to the relevant stimuli, that all reproduce satisfyingly the collective patterns. It is then theoretically established that two of these models, despite being clearly distinct in terms of individual behaviors, cannot be discriminated using collective scale observational data, whatever their accuracy and amount. We then address the methodological questions associated to the design of additional individual-based experimental protocols, as well as the use of the corresponding data for model validation and inversion of free parameters. A sequence of methodological steps is proposed and practically illustrated using the same object clustering example and the expected benefits are discussed.

## Results

### Identical Collective Pattern Dynamics with Distinct Individual Behaviors

#### Object clustering in *Messor sancta*

**Figure 1:**
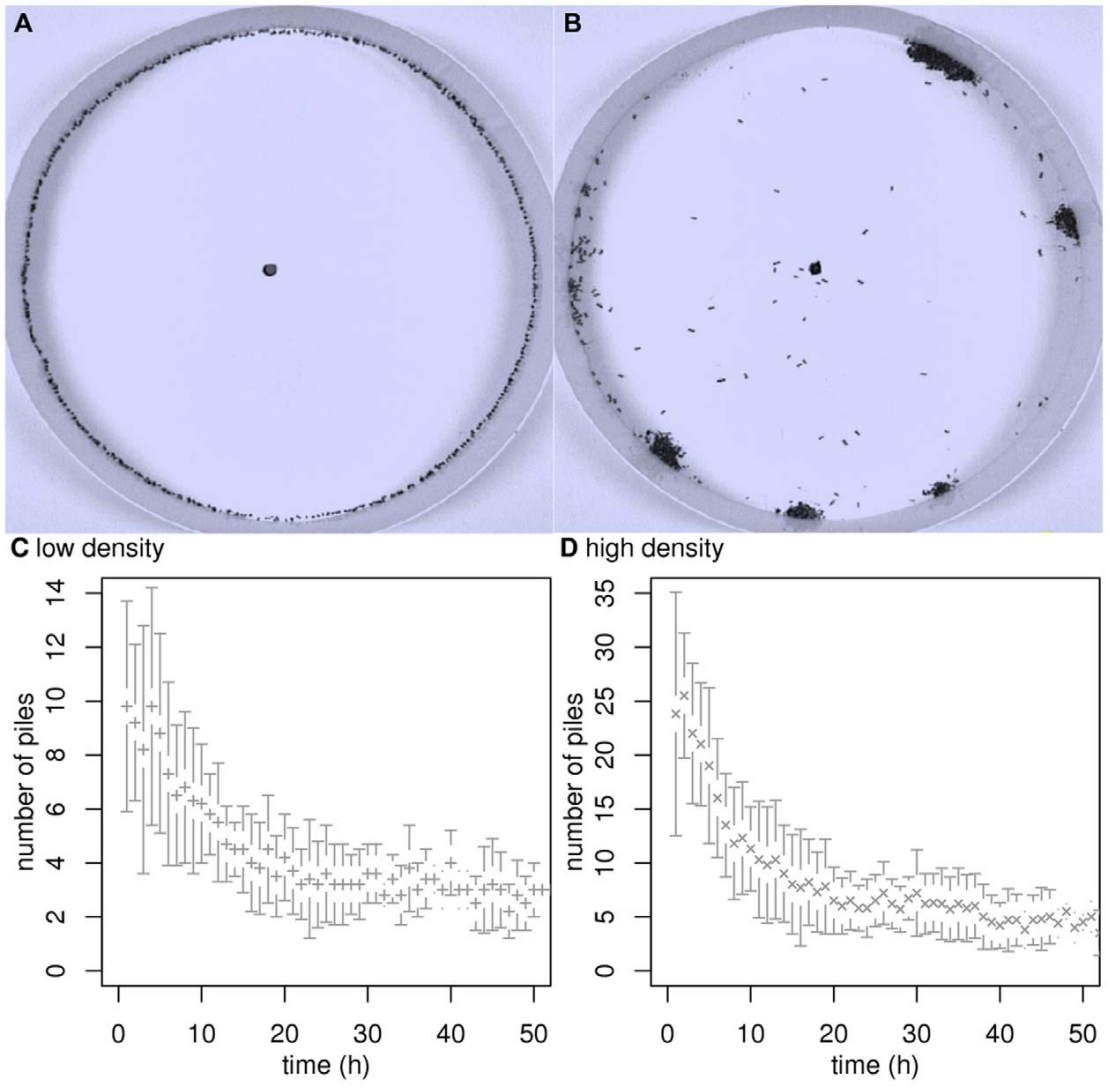
Clustering experiments. Objects are uniformly distributed along the border of a 50 cm diameter circular arena. Ants enter the arena spontaneously from below, mounting along a wood stick through the hole in the arena center. Two different initial one-dimensional densities are used: 127 and 255 objects per meter. The duration of each experiment is 50 hours. Fig. A and Fig. B correspond respectively to the beginning and the end of a high density experiment. Fig. C and Fig. D display the time series of the number of piles (mean 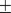 s.d.) for the low and the high density experiments, respectively. Piles are defined as follows: Two neighboring objects are considered to belong to the same cluster if the distance between them is less than 1 mm. A cluster constitutes a pile if it contains at least 6 objects. In [41] another circular arena with a 25 cm diameter was also used (with the same low and high initial densities). For the purpose of the methodological illustration in the first part of the results section only the large arena is used, but the model built in the second part of the results section is compatible with all observations, including the ones in small arenas (see Fig. 6).

We will tackle all methodological issues with the help of a simple example of collective animal behavior: object clustering in the ant *Messor sancta* [41] (see Fig. 1). In this example, the objects clustered by the ants are corpses of their dead conspecifics (note that social insects, and in particular ants, are also known to build clusters of many other kinds of objects, e.g. brood [42], seeds [43], [44], sand pellets [45] and leafs fragments [46]). The spatial structures result from dynamics in which the behavior of each ant is indirectly influenced by its conspecifics by way of the result of their activity. One of the features of this collective phenomenon is that the behaviors can be studied under controlled laboratory conditions at two different scales, that of an individual and that of the resulting clusters. The experiments reported in [41] were carried out in circular arenas (see Fig. 1A). As the objects (the ant corpses) are initially distributed homogeneously along the arena wall and as the ants exhibit a strong tendency to follow the inner wall of the arena, the whole clustering process takes place within a small band along these walls. When the ants are given access to the arena, they preferentially move along the walls, pick up objects at some places and deposit them elsewhere. After a few hours several small piles, regularly distributed in space, can be observed. The bigger piles grow at the expense of the smaller ones, that have completely disappeared by the end of the experiment (see Fig. 1B). Therefore the measured mean number of piles (see Fig. 1C and Fig. 1D) rises first up to a peak after ca. 2 hours (about 11 and 25 piles, respectively, for the small and high object density configurations), then decreases and finally reaches a quasi-stationary number at the end of the experiment (after 50 hours, about 3 and 4–5 piles, respectively).

All the reported attempts to model such object clustering behaviors start with the assumption that no significant chemical marking process is at work (no pheromone deposition), that direct interactions between individuals play a neglectable role and that ants move according to a constant speed diffusion random walk [31], [32], [41], [47]. Clustering is then the result of a competition between the homogenizing potential of diffusion and the stimulation / inhibition of object picking up and deposition by the perceived local density of objects 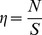, where 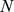 is the number of objects in a perception area 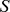 around the ant.

#### Three model ingredients and their cognitive significance

Even if we take these modeling choices for granted (their discussion is not the purpose of the present contribution), numerous questions remain widely open. For the purpose of a brief illustration, we will play in this section with three behavioral concepts: we will question the influence of the perceived object density, the importance of inter-individual variability, and the role of individual temporal correlation.

The influence of the perceived object density targets the shape of the stimulus-response function, namely what the ants react to. As far as corpses are concerned, Anderson [47] explicitly asked: “But what if an ant regards a group of two or more ants as a processed pile and perceives a single dead ant as something qualitatively different – perhaps as an unprocessed ant that just happened to die on that spot?”. It is generally admitted that deposition behavior is favored by the local object density, but does it also affect the picking up behavior? In such a context, the model interpretation would be about whether there is some distinct information processing dedicated to object perception or not. Object density could well favor local deposition simply due to mechanical constraints because it is harder to carry an object through a cluster of objects. It is known for instance that ants can build walls of rocks by just reacting to obstacles [32] or mounds by randomly depositing their load [48], in which case there is little need for sophisticated cognitive processes. Yet, some other species display an added complexity for nest wall building, with individuals coordinating the choices of material they fetch on independent forays [49].

The inter-individual variability would refer to different activity levels among workers, which is well-known in social insects as division of labor [50], [51]. Division of labor and task partitioning in social insects are often cited as major features of their ecological success as they are reputed to increase colony efficiency because specialized workers can become superior in performance for their task [52]–[54] (although this is controversial [55]). In the context of necrophoric behavior, it can also allow the colony to keep waste-workers and waste piles away from vital resources (e.g. fungus garden) in order to reduce contamination risks [56], [57]. The division of labor can stem from genetic bases [58], [59] but strongly depends on colony size and needs as well as to workers’ age [51], [60], [61] and physiological state [62]. Such differentiated objects handling activity was introduced in the model by assuming inter-individual variability, i.e. their picking up and deposition statistics depend on an activity level 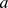 that is constant in time but differs for each ant. The activity level could for instance be related to age or any kind of genetic or epigenetic factors. Inter-individual variability could play a significant role if the activity level distribution is wide enough for significantly modifying the picking up and deposition statistics compared to that of a population with all individuals reacting the same.

Finally, individual temporal correlation refers to some kind of memory effect, i.e. the behavioral decision (e.g. deposition) is affected by the time elapsed since the last behavioral decision (e.g. picking-up). Such reference to a memory effect has been recently suggested for the necrophoric behavior in *Myrmica rubia* [63]. A memoryless behavior would not exhibit temporal correlation, i.e. the behavioral response at time 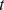 would only depend on what is perceived by the ant at that time, and this is generally assumed implicitly in behavioral models based on stimulus-response functions. Note that such a memoryless model would not exclude learning processes or individual experience for specialized individuals [64], [65] as they could still be accounted for by adapting the parameters of the stimulus-response function itself. However, since we are interested only in memory effects of the same magnitude as the time scale of the collective phenomenon, we rather chose to test a true memory effect modelled by a decreasing propensity to pick-up or deposit as the time elapses: the longer the time an ant has been carrying an object, the less reactive it will be to local density, as if it would explore longer for a higher density to compensate for the increasing effort made to reach it.

#### Distinct models leading to comparable (or identical) clustering dynamics

**Figure 2:**
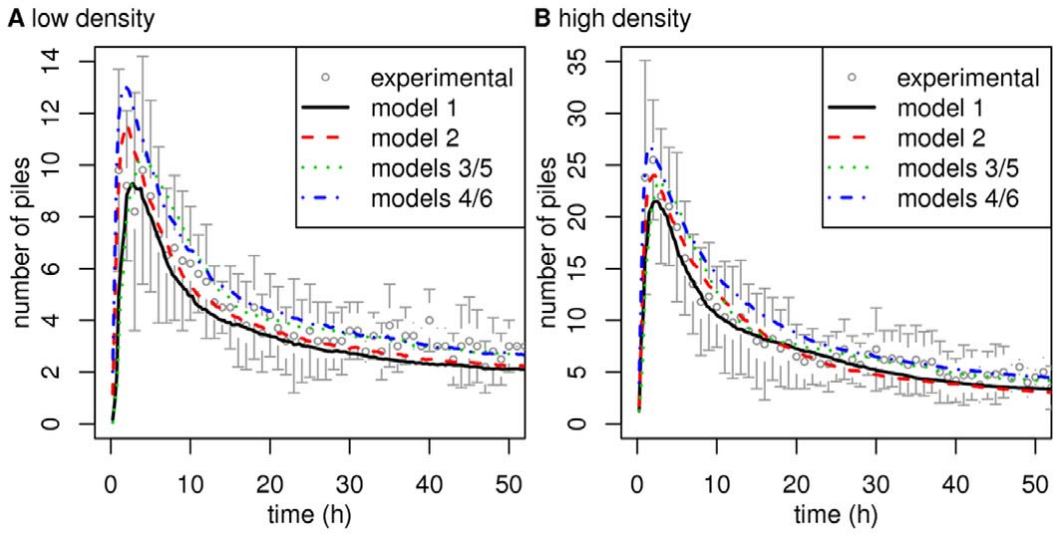
Clustering dynamics predicted by the six different models. Figs. A and B indicate the time series of the number of piles for models 1–6 compared to the experimental data (mean 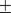 s.d.) in the low and high density settings, respectively. The predictions of all the models are compatible with the experimental observations. Moreover, the predictions of model 5 are rigorously identical to those of model 3, and the predictions of model 6 are identical to those of model 4.

The Methods section indicates how these three behavioral components are combined to produce six different models. The point is that all six models allow functional fits that make them quantitatively compatible with the available data on clustering dynamics (cf. Fig. 2), although they strongly differ in terms of cognitive implications as far as the interpretation of picking up and deposition behaviors are concerned.

At this stage, further investigations to identify the most relevant individual model would most commonly start by looking for more detailed quantitative measurements on the basis of the same observed clustering dynamic. In addition to the temporal evolution of the number of piles, one may analyze the distribution of inter-cluster distances, of cluster sizes (number of clustered objects), cluster dimensions, etc., hoping that these distributions could be characterized with enough statistical accuracy to exclude some of the six models. However, one may easily think of numerous cases in which the required accuracy would be so high that no experimental protocol would allow it to be reached. It is for instance impossible, from macroscopic observations of gaseous properties, to discriminate between two molecules having different collision cross-sections if these different “interaction behaviors” lead to the same values of viscosity and conductivity. However, in our illustration example this point can be made even more explicit than in the frame of the asymptotic theory, in particular without any restriction to a macroscopic limit: we proved indeed theoretically that it is strictly impossible to discriminate between models 3 and 5 (picking up behavior independent of the perceived object density 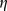), or between models 4 and 6 (picking up behavior inhibited by 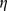), whatever the amount and the accuracy of the information acquired from the observed object displacements, in particular whatever the observation scale. The extended proof is given in the Methods section, but its principle is quite simple:

- The picking up probability is the same for each individual ant in models 5 and 6 (no inter-individual variability), whereas it depends on the activity level in models 3 and 4 (inter-individual variability), but the activity level distributions are such that the population average value of the picking up probability of model 3 equals that of model 5, and that of model 4 equals that of model 6. Each object is therefore picked up with the same temporal statistics within each model pair.
- The probability of still carrying an object decreases exponentially as a function of time in models 3 and 4 (no temporal correlation), whereas it decreases non-exponentially in models 5 and 6 (temporal correlation), but the survival curves in models 5 and 6 are such that, when integrated over the whole population, they lead to the same deposition statistics as those of models 3 and 4 respectively.
- For each object, the picking up and deposition statistics resulting from the whole population are therefore rigorously identical within each model pair, meaning that the models cannot be discriminated by the observation of object displacements alone, whatever the accuracy level.

The conclusion would be the same even if the living ants could be followed and their behavior statistically analyzed during the clustering experiments, provided that a single ant could not be tracked long enough for its individual statistics to be characterized independently from the other ants. Otherwise the activity level distribution could indeed be observed and models could be discriminated. The crucial alternative is therefore the access or not to the details of the individual ant behavior.

### A Methodological Approach to the Identification of Individual Behavioral Rules

#### Individual versus collective scale modeling

The preceding examples were meant as an introduction to the methodological questions raised by the objective of not only modeling the collective dynamics, but also learning about the involved individual behaviors with enough details and confidence to allow fruitful contributions to cognitive and physiological biological research. The literature emphasizes the fact that details of the individual behaviors are widely irrelevant to the understanding of numerous emerging collective behaviors, which legitimates that solid theoretical conclusions can be drawn without deep references to the physiological and cognitive abilities of the considered species. But the same fact translates into strong practical difficulties as soon as “details”, from a collective point of view, may correspond to such significant biological differences as with or without inter-individual variability and with or without short time memory usage. We even formally established (see above) the existence of configurations that rigorously exclude the discrimination of two different biological interpretations from observations of emerging structures, whatever the temporal and spatial observation scales: the emergence statistics can be strictly identical with two behavioral models that are very distinct in terms of cognitive implications. We therefore face the question of looking at other observables than those defined for the purpose of collective modeling, using the same available experimental data, or implementing new experimental protocols specifically designed for the purpose of individual behavior modeling.

First of all, it may be useful to note that the distinction is quite subtle. There is nothing like a pure collective scale reasoning on one side, versus pure individual scale reasoning on the other. The question still remains the understanding of the collective behavior, which means that:

- we are only addressing the components of the individual behavior that impact the collective features,
- the individual model is only fully validated when it can be shown that the corresponding perceptions and actions are sufficient to reproduce the addressed collective patterns.

The only difference from a pure collective scale reasoning is that we try to add the argument that the identified individual behavior is not only sufficient to reproduce the collective dynamics, but that it is indeed at work in the considered species. There could be ways to fully distinguish individual studies from collective ones if it were established that collective modeling would systematically lead to the identification of effective parameters that would summarize the effects of a potentially wide diversity of possible individual features. Individual behavior modeling could therefore take the effective parameters as their unique basis and the question would only be to understand which one, among all possible behaviors, is responsible for the observed effective parameter value in the considered species. This would be a complete parallel with, for instance, gaseous kinetics where fluid dynamics deals, at the hydrodynamic limit (the collective scale), with the question of how effective parameters such as viscosity and conductivity impact the flow dynamics, whereas quantum molecular physics deals, separately, with the question of how specific molecular structures and properties give rise to the observed viscosity and conductivity values. This may appear to be meaningful as far as bird flocks and fish schools are concerned, but the preceding object clustering examples are sufficient to demonstrate that individual models that are indistinguishable at the collective scale do not systematically refer to identical effective parameters in a common collective model. A strict separation between individual and collective studies is therefore hard to maintain.

Altogether: i) we need to go further than designing a valid collective model; ii) the additionally required information may not be accessible from collective observables; iii) the collective experimental data remain the material used for the final validation of the proposed individual model. At this stage, two approaches can be retained or combined: either the same experimental protocol (or field observations) are used and new observables are defined with the objective of closely characterizing the individual properties, or independent protocols are designed. We concentrate hereafter on the design of independent protocols, which may be considered as the easier approach (when possible) in the sense that: i) there is no ambiguity associated to the fact that the same data are used both for parametric quantification of the retained individual model and for final validation in terms of emerging collective features; ii) the involved different behavioral components can be addressed separately by designing experimental protocols dedicated to the characterization of a single component. We can think for instance of separate dedicated protocols for the movement of ants, for picking up and for deposition, whereas the initial object clustering experiments involve the three actions simultaneously. We argue hereafter that when designing these protocols, strong benefits can be expected from the effort of distinguishing the following successive methodological steps:

1. enunciation of a behavioral model (or several alternative ones);
2. model translation into fully quantitative terms involving the choice of well defined state variables and stimuli;
3. validation and parametric inversion of the behavioral components that can be characterized independently of all other components, or one by one in an adequate sequence;
4. coupled validation and parametric inversion of the remaining components;
5. confrontation of model predictions to the available observational data collected at the collective level.

The end of the present section is devoted to the explicit development and the illustration of each of these steps successively.

#### 1. Model enunciation

The qualitative discussion of the behavioral model is the step at which most of the biological reasoning takes place. The term *model* is therefore to be understood as an argued representation of the individual behavior as far as its influence on the collective dynamics is concerned. We will come back to this point in the discussion section, in particular to the fact that experimental protocols aiming at the detailed characterization of individual behaviors are designed with the idea of validating or invalidating one or several alternative behavioral models, and that an explicit statement of such initial orientations helps clarifying the subsequent cognitive and physiological debates. Here we only illustrate this first step with the example of [41] in which a literature review and preliminary experimental explorations led to the following model enunciation:

- Individuals have identical behaviors (no inter-individual variability).
- Direct inter-individual interactions play no role in the object clustering process.
- Indirect inter-individual interactions by way of pheromone deposition play no role in the object clustering process.
- Ants are always moving, speed changes play no role in the object clustering process and actions such as direction changes, picking up and deposition are instantaneous.
- Thigmotactism is so strong that ants remain strictly in a narrow band close to the arena border.
- Objects never overlap.
- Objects are only perceived in the immediate vicinity of the ant, and this perception corresponds to an antenna contact and/or a visual perception, both having similar ranges (of the order of a few millimeters).
- Object perception leads to the estimation of the local object density via an indirect measure of the number of objects in the perception area.
- Although object perception is very local, it could be claimed that the individual has access to more complete information concerning the object field if it kept a memory of the objects it encountered along its trajectory: the retained model states that such a memorization process is not at work and that, at each instant, the ant behavior is only influenced by current local perception.

Some of these statements do not appear explicitly in the four pages format of [41], but are all extracted from the broader, partially unpublished, underlying investigations.

#### 2. Quantitative translation

**Figure 3:**
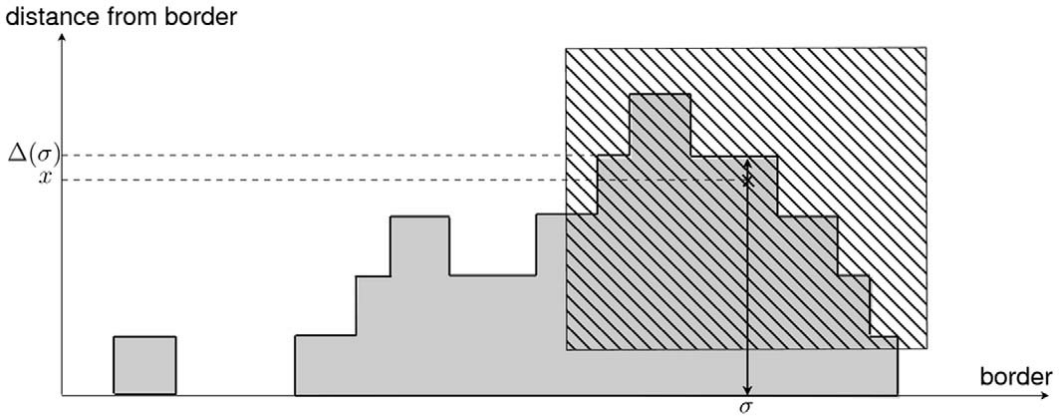
Definition of the object perception stimulus. The grey area represents the objects distributed along the arena border and the dashed square the perception range of an ant at perimetric abscissa 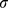 and distance 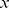 to the border of the arena. The dashed grey area represents the fractions of the objects perceived by the ant (which corresponds to 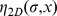 in the text). When defining the perception stimulus at perimetric abscissa 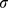, without knowing the distance to the border 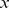 (one-dimensional modeling), 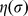 is defined as the average of all values of 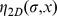 when 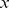 is uniformly distributed between 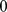 and the distance 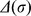 of the external side of the farthest clustered object.

Complete model translation into quantitative terms requires arbitrary choices to be made for state variables and stimuli that cannot be deduced as direct consequences of the model once it has been enunciated. *Arbitrary* refers here to cognitive reasoning: if there were cognitive motivations for the quantitative definition of some state variables and stimuli, they should be part of the model itself and should be enunciated and justified in the preceding step. This does not exclude motivated arguments toward the choice of a given state variable (or stimulus) rather than another meaningful one, in particular for practical reasons related to experimental observation or to any forthcoming formal derivation. Our object clustering example provides various illustrations of the typical meanings of such required arbitrary choices. Quantitative translation of ant movement is the simplest of these illustrations: the only enunciated related properties are that ants remain close to the circular arena border, that speed changes are insignificant, and that direction changes are instantaneous, which is translated into the fact that ant locations are reduced to a lineic abscissa along the arena perimeter and that ant velocity is either clockwise or counterclockwise oriented, with a constant speed. This means in particular that the details of the thigmotactic behavior are left aside and that only its overall effects are quantified. On the experimental level further detailed definitions were required in order to measure the abscissa and the orientation of each ant. A first ant location was identified using the center of the head and was projected on the perimeter; the orientation of the movement was then defined using the difference between the successive abscissa values corresponding to two successive frames, etc.; but this belongs to the measurement protocol and not to the model translation itself. More subtle but very well established is the translation of the fact that ants do not use any memorization process for the considered actions. We already mentioned the quantitative translation of this assumption in the preceding section when describing the model examples in which the assumption was made that no temporal correlation occurred. The fact that the enunciated model excludes any significant effect of memorization, combined with the assumption that direction changes, picking up and deposition are instantaneous, translates indeed into the statistical property that whatever occurs after a given time 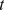 is statistically uncorrelated to any preceding action or perception. This allows Markovian formulations to be used, which leads in the present case to exponential survival probabilities such as those of Tab. 1 for the states of traveling clockwise, traveling counterclockwise and carrying an object. Practically speaking, this means that the actions of changing direction and depositing an object are fully quantitatively described by two functions of the object perception stimulus 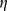: the direction change frequency 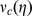 and the deposition frequency 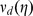, or alternatively the average time before direction change (the direction change mean free time) 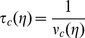 and the average time before deposition (the deposition mean free time) 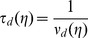. An essential point here is that no arbitrary choice was made for this translation: frequencies can be preferred to mean free times or vice versa, but the translation of a memoryless behavior with instantaneous actions is fundamentally unique, whereas the level of detail that was retained for the quantitative representation of location and movement was very much arbitrary. Let us give a last translation example with the definition of the object perception stimulus 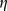 in [41]: this definition is arbitrary but is motivated by the objective of simplifying formal derivations, in particular in view of a linear stability analysis of the initial phase of clustering. Object perception is widely unknown or is only accessed very indirectly. A consequence is that it is difficult to make a meaningful choice for 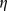. Do we have to account for visual blocking of an object by a closer one? Should we opt for a strict counting of the number of objects in the perception area, or should we think of an indirect measure of the local object density? What is the shape of the perception area? Again, if one of these questions could be addressed with a satisfactory level of confidence, the answer would be given during the model enunciation phase. As such answers are not available or were not available at the time of [41], the freedom in the translation process is large and the choices must be motivated by other types of reasoning. The final, reported choice was the following: The perception area is a square of size 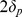 around the ant location. One side of the square is parallel to the arena border, whose curvature is assumed negligible for such local perception reasoning. Each object has a square shape of size 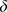. One of its sides is either in contact with the arena border or in contact with the external side of another square object, which allows that clusters extend several times 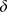 away from the arena border (see Fig. 3). A first definition of a perception stimulus 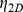 is then the sum of all the perceived object fractions divided by the perception area, that is to say 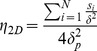 where 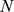 is the number of perceived objects and 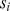 is the surface of the 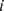-th object that lies within the perception area. This definition is strongly related to the measure of a surface object density. The fact that perceived objects that lie across the perception area border contribute via a fraction of their surface ensures that this perceived surface density is continuous in space. Such a choice had three significant practical consequences:

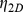
 could be used in studies where ants were followed along their detailed two dimensional trajectories, independently of the question of simplifying the final representation of ant location and velocity in terms of perimetric abscissa and clockwise or counterclockwise orientation.
- The extension of the work reported in [41] toward two dimensional object clustering modeling was straightforward when attempting to analyze clustering experiments for which objects were initially spread uniformly on the total surface of the arena instead of being aligned along the perimeter.
- The fact that this surface density is continuous allowed the later meaningful use of macroscopic reacto-diffusive approximate models for theoretical analyses of cluster emergence and cluster selection.

But these practical advantages have the drawback that 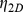 cannot be evaluated using the retained state variable for position (the perimetric abscissa). Indeed, two ants having the same perimetric abscissa 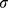, but located at two different distances 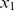 and 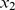 to the border, perceive two different values 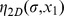 and 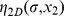 of the surface object density. It was finally chosen to define the object perception stimulus 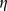 at a given perimetric abscissa as the average value of 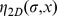, for a uniform distribution of 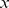 between 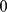 and the distance 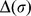 of the external side of the farthest clustered object (see Fig. 3):
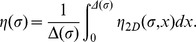



With this definition, 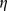 can still be interpreted as a surface object density and it inherits the continuity features of 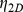 which allows the efficient use of approximate reacto-diffusive models, now for the one dimensional analysis of cluster emergence and cluster selection along the perimeter. These features were intensively used in the research reported in [41], in particular via linear stability analysis of reacto-diffusive forms of the one dimensional model that predicted a critical value of the initial object density below which no clustering occurred, a property that was later confirmed experimentally and was interpreted as a strong validation of the overall modeling approach. Altogether, a quite complex choice was made for the definition of 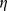. Some of its features are quite arbitrary from a cognitive point of view, but they rigorously reflect the two essential cognitive properties enunciated in the perception model: its limited range and its graduality. This choice was also guided by the subsequent formal derivations, leading to experimental explorations and theoretical conclusions that would have been difficult to reach with less refined definitions.

#### 3. Uncoupled validations and parametric inversions

**Figure 4:**
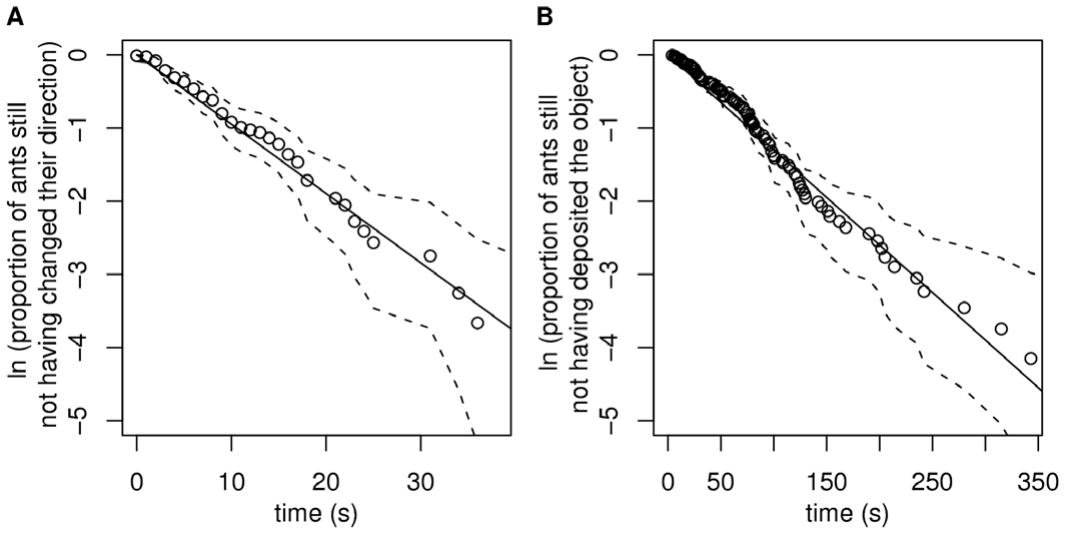
Parametric inversions for the direction change and deposition frequencies in the empty arena. A) The survival curve of the proportion of ants still not having changed their direction (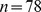 trajectories) is compatible with an exponential fit 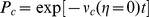, validating the memoryless and instantaneous turn assumptions and leading to the direction change frequency 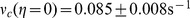 (mean 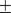 s.e.), 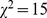, 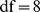, 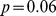. B) The survival curve of the proportion of ants still not having deposited the object they loaded at time 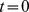 in the empty arena (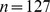 trajectories) is also compatible with an exponential fit 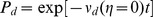 and leads to the deposition frequency 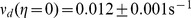, 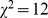, 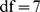, 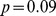. The black line represents the exponential fit and the dashed lines the 95% confidence interval. The 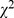-values correspond to a chi-squared test for goodness of fit as described in [72] (pp. 131–137).

Once the model is fully translated into quantitative terms, dedicated experimental protocols can be designed in order to validate successively each of the behavioral components of the model and determine the remaining free parameters (e.g. the perception range 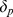 and the object size 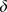 in the preceding translation examples) as well as the remaining free functional dependences (e.g. the dependence on the object perception stimulus of the direction change frequency and the deposition frequency, that is to say the functions 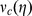 and 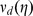). At this stage, a detailed biological knowledge of the considered species is again required in order to evaluate the feasibility of such dedicated experiments and to make sure that the behaviors in the intended experiments will be identical to those at work in the initial collective conditions. In [41], the first of these experiments consisted in introducing a single ant in the empty arena, following it during its thigmotactic behavior along the arena border and measuring the successive time intervals spent in the clockwise or counterclockwise direction. This allowed to check that the corresponding survival curve was indeed exponential, which validated the memoryless and instantaneous turning assumptions, and to measure the direction change frequency as the inverse of the average value of the measured time intervals. Very similar is the experiment in which a single object carrying ant was followed in the empty arena leading to the evaluation of deposition frequency. Fig. 4 illustrates the kind of fitting qualities that are typical of such validation and inversion exercises in the most successful cases.

#### 4. Coupled validations and parametric inversions

But less favorable conditions than those described in the previous paragraph (3.) are very commonly encountered as soon as the components of the behavioral model cannot be easily isolated. It is indeed often concluded that even the most sophisticated experimental protocols lead to quantitative measurements that correspond to the combination of several elementary behaviors, which means that the validation exercise is likely to be much less convincing and that one may expect to find several distinct solutions in the inversion process. Somehow, as soon as dedicated experiments address high levels of complexity (e.g. when several behaviors are involved in an intricate manner), we are back to the methodological difficulties associated to the collective observations: several distinct models can be convincingly fitted to the available observations. In most cases this difficulty is unavoidable, but attempts can be made to lower it as much as possible: experiments can be repeated in order to reduce statistical uncertainties, new protocols can be designed to help distinguishing between several identified inversion solutions, and, maybe more importantly, the inversion procedure itself can be gradually modified so that the components of the model are discussed separately, even if they cannot be fully isolated. When such attempts are successful, the complexity level can be significantly reduced and, instead of only presenting the final result as *one* possible solution of the simultaneous inversion of all the involved components of the behavioral model, the elementary behaviors can be addressed one after the other, even if this requires to make simplification assumptions. The solution is then presented as *one* proposed solution based on arguments that are open to debate and can be validated or rejected using additional experiments. The point that we try to make here is that coupled inversion procedures are not pure technical exercises, except when so many complementary experimental data are available that only one solution is acceptable. The existence of a solution only confirms that the model is meaningful considering the statistical uncertainties of the available observations. Retaining *one* among the commonly large number of possible solutions for free parameters and free functional dependences is always a choice, and is therefore open to further biological discussions and further dedicated experiments.

The above presented validation and parametric inversion examples concerned the particular case where no object was perceived and led to the estimation of direction change and deposition frequencies 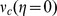 and 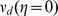 in an uncoupled manner. But the next reported validation and inversion exercise in [41] involves simultaneously ant movement, object picking up and object deposition. Without entering into all the details of this coupled inversion example, let us use it to illustrate the kind of procedures that may lead to the identification of one among the multiple inversion solutions. The corresponding experimental protocol consisted in the artificial gathering of objects into clusters of controlled shape and size. The measured statistics were those of the result of the overall interaction with the cluster, in particular the probability that the ant leaves the cluster in the object-carrying or non-carrying state, knowing its state when first perceiving the cluster. During the time of such a cluster encounter:

- the ant perceives continuously varying values of the perception stimulus 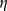 (weak values when first perceiving the cluster and strong values when in the center of the cluster);
- the ant may change several times its orientation and may successively deposit its object and pick up another one several times before leaving.

**Figure 5:**
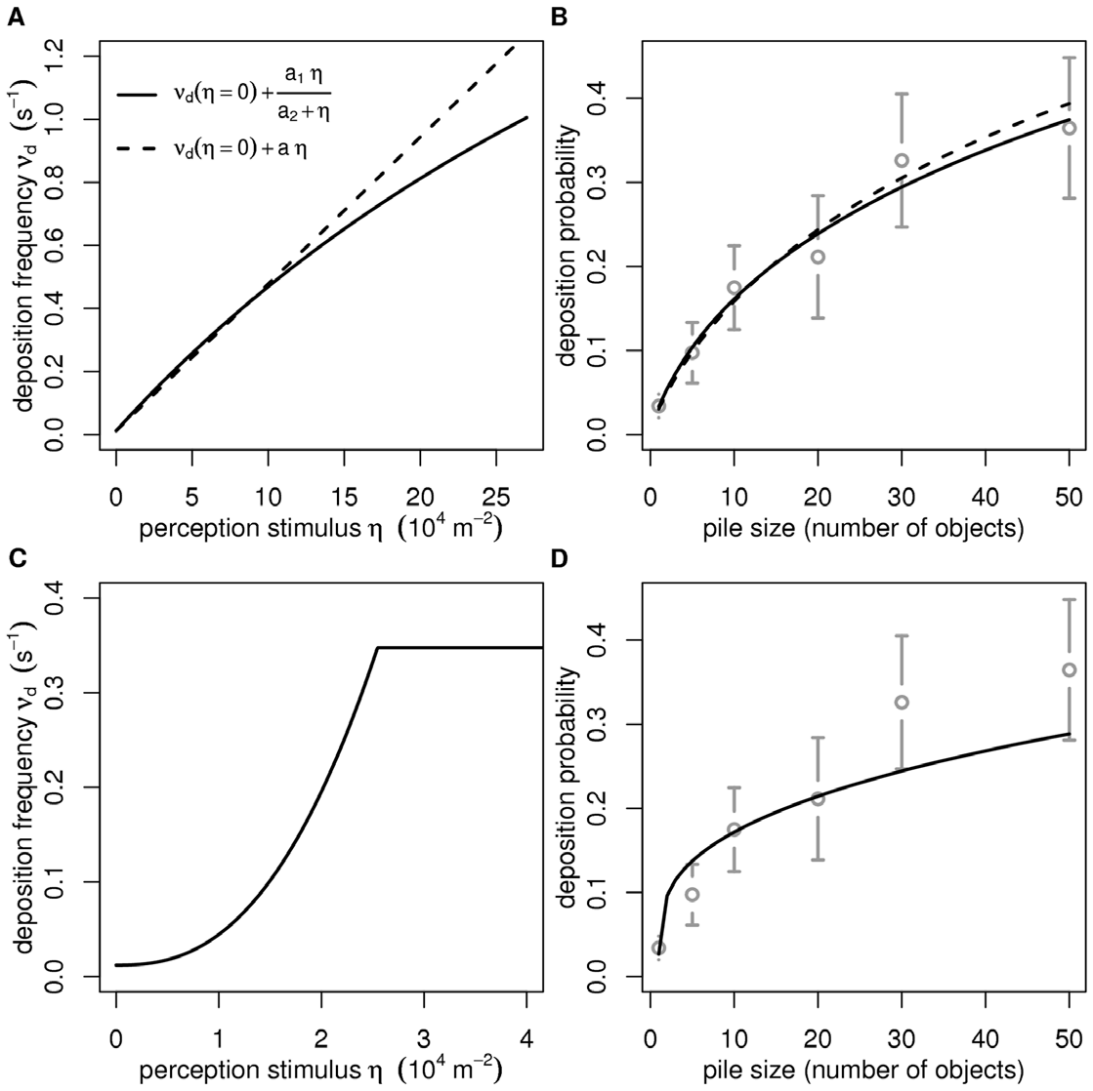
Parametric inversion for the deposition frequency. A) Functional dependence of the deposition frequency on the perception stimulus. B) Fit of the deposition frequency to the experimental deposition probabilities (mean 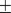 s.e.) on clusters of several sizes. The plain curve in A corresponds to the formal dependence retained in [41] (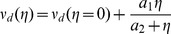, with 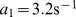 and 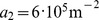, 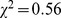, 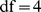, 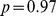). The dashed curve is a linear fit (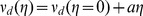 with 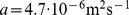, 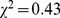, 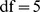, 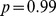), very close to the previous formal dependence for small values of the perception stimulus. C) An alternative functional dependence of the deposition frequency on the perception stimulus, in which the deposition frequency is very low for small values of the perception stimulus and constant for values higher than a threshold corresponding to two objects entirely in the perception area. D) Adjustment of C to the experimental deposition probabilities (
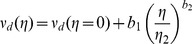
 if 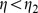 and 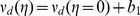 else, with 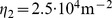, 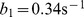 and 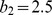, 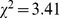, 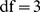, 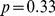). The grey circles correspond to the experimental data (mean 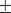 s.e.) and the 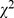-values to a weighted least squares procedure.

This means that the inversion exercise addresses the whole 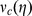 and 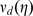 functional dependences, and involves them simultaneously together with the picking up probabilities. The first choice that was made consisted in attempting to find an inversion solution in which the movement was not affected by the perceived object density, which translates into the fact that the direction change frequency is fixed to 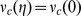. This choice was motivated by qualitative observations indicating that the movement of ants is little affected by the presence of objects. But this indication remained highly questionable as it was not confirmed by quantitative measurements and was not sustained by any kind of previous behavioral knowledge (otherwise it would have been part of the model enunciation): it was only indirectly and weakly validated by the fact that inversion was still possible, fitting only the other remaining free functional dependences. Another choice took the form of an a priori assumption that could be validated a posteriori: it was assumed that object picking up probabilities were so small that when an ant deposits an object on a cluster, it systematically leaves the cluster without picking up a new object. This allowed to decouple the deposition from picking up behaviors when analyzing all observations corresponding to an ant carrying an object when first encountering the cluster: in such cases the ant could either leave the cluster without depositing, or deposit the object and leave the cluster, but no picking up was involved. Furthermore, as movement was already fixed, only the deposition frequency remained unknown and the inversion became of the uncoupled type. The dependence on 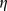 of the deposition frequency could therefore be studied independently. Once the function 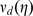 known, the other observations could be employed (i.e. those in which the ant was free when first encountering the cluster) to study the picking up probabilities, using the now fixed movement and deposition behaviors. Then the a priori assumption (no picking up after deposition) could be validated by re-processing the deposition inversion (without neglecting picking up, using the obtained picking up probabilities) and checking that the same 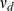 dependence was found. The inversion was therefore held step by step: movement was fixed arbitrarily, deposition was characterized using a subset of the observations for which picking up could be assumed to play a neglectable role, and (with fixed movement and deposition statistics) picking up was then characterized as the last remaining behavior using the fully coupled observations. A last essential point is the way functional dependences were inverted. Characterizing the deposition frequency, for instance, means indeed that a full functional dependence needs to be determined. There are many approaches to this difficult question in the inverse problems literature, but in all cases a formal dependence is fixed in advance with free parameters to be fitted. When a large amount of accurate experimental data is available, the formal dependence can be very little constraining, as when using high degree splines or wavelets, but when the available observations are sparse and statistically quite uncertain, the formal dependence is a significant choice. In [41] the deposition frequency was given the following shape:
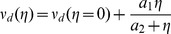
in which 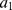 and 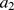 were free parameters. A least squares procedure was then used to check that this shape was compatible with the observed cluster interaction statistics, and to evaluate 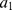 and 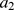. Fig. 5 illustrates the result of this least squares procedure and also displays two alternative results obtained with distinct formal dependence choices, in order to illustrate the degree of freedom that one faces under such validation and inversion conditions.

#### 5. Confrontation to collective observations

**Figure 6:**
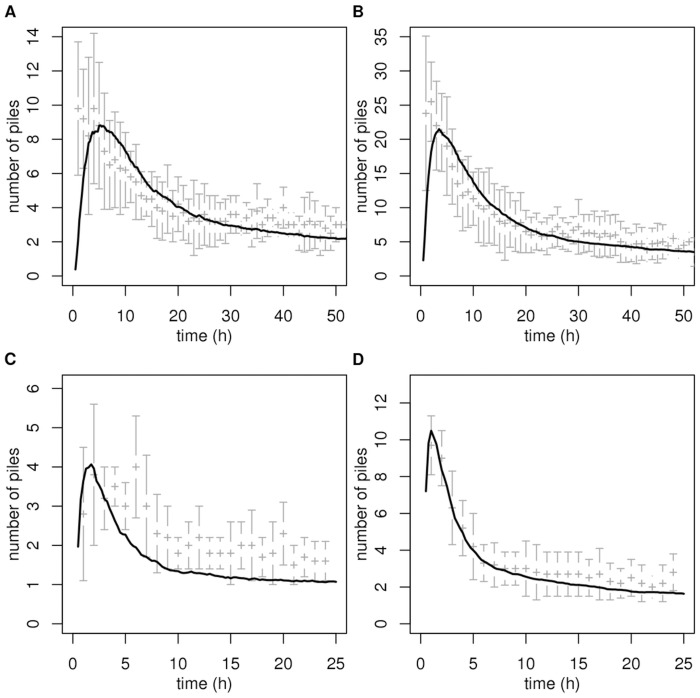
Comparison with experimental data of the collective dynamics predicted by the finally retained model (that of the dashed curve in Figs. 5A and B). Figs. A and B correspond to the experiments in the big arena, and Figs. C and D to those in the small arena, with low and high object densities, respectively (see Fig. 1). The grey + correspond to the experimental data (mean 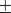 s.d.) and the plain curves to the model predictions. The fitted number of ants in the arena is 50.

Whatever the level of confidence of the overall inversion procedure, the last step consists in confronting model predictions to observations of the collective behavior we actually want to explain. From the start, we only discussed methodological approaches in which the behavioral model could be entirely validated (and the free parameters determined) using dedicated experiments, meaning experiments that are independent of the initially addressed collective scale experiments. When this is the case, no further fit is required before running the final simulations and checking that the emerging collective behaviors compare satisfactorily with observations. This independence is a strong guarantee against the remaining weaknesses of the preceding model validation procedures and more generally against the difficulties illustrated in the first part of the results section: the more parameters remain to be fitted using the collective scale experiments, the less one is indeed protected against the risk of matching the collective behaviors with little confidence in the cognitive and physiological pertinence of the corresponding behavioral model. The object clustering example that we used for illustration throughout this methodological description led to the very satisfactory final simulations reported in Fig. 6, with only one single additional parameter fit: the number of ants in the arena, that was very much fluctuating during each experiment, and which only influenced the time scale of the clustering process, but not the shape of the temporal evolution of the mean number of clusters.

## Discussion

What are the expected benefits of distinguishing the above presented methodological steps in an explicit manner? The object clustering example used throughout this paper should already be sufficient to argue that a first benefit is a clear listing of all those among the reported choices that are related to biological and cognitive reasoning and can be discussed as such (from the model enunciation to the design of experimental protocols and even to parts of the inversion procedure), in opposition to technical acts that are only criticizable in terms of formal or statistical pertinence and rigor (from quantitative model translation to statistical inversion). An immediate consequence is the highlighting of both the strongest and weakest components of the proposed behavioral model. In the context of collective animal behavior, no model proposition will ever be reported if it does not at least partially reproduce the collective scale observations. Collective scale predictive power is therefore a minimal requirement but is not sufficient in the above defined context. Strengths and weaknesses of elementary behavior representations are therefore judged in terms of individual scale predictive power and their cognitive significance. The less solidly established behaviors will hopefully be further investigated experimentally or will motivate further explorations of the literature on cognitive and behavioral processes, but the most established ones, when made explicit, are likely to serve as starting points for other biological researches, even outside the research field of collective animal behaviors. More broadly speaking, some kind of clarification effort is undoubtedly required if a modeling attempt claims to be more of a contribution to cognitive research than the six model examples of the first part of the results section. However, as we tried to point out all along the present paper, the distinction is commonly very subtle. First because it is hard to design experiments that allow to address the elementary behaviors independently or successively; second, because the number of possible experimental replications is often quite limited, the sample sizes are small, the statistical inversion procedures are weakly constrained, which means that even when making strong efforts toward explorations on the individual scale, multiple inversion solutions are available and the criticisms formulated in the second part of the results section apply here also, at least partially.

An example of strong validation mentioned above was that of the temporal decorrelation assumption in [41]. More precisely, both the temporal decorrelation assumption and the absence of inter-individual variability assumption were strongly validated. Although we did not mention it in the results section, the experimental results reported in Fig. 4 were indeed obtained by gathering the data corresponding to several tens of distinct ants. The observed linear shape of the logarithmic scale survival function over several decades is therefore a solid guarantee that both memorization and inter-individual variability play a neglectable role. But we saw that the conclusions reached concerning the influence of the perceived object density on the movement, picking up and deposition actions are much weaker. We already illustrated in Figs. 5A and B the fact that two different functional forms led to satisfactory inversion solutions. These two forms are close in most of the relevant 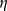 range, where 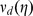 increases linearly. However Figs. 5C and D display a third inversion solution where 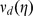 is quasi constant for small values of 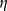 until enough objects are perceived for a positive feedback to appear. The only motivations for not retaining this possible inversion solution are that no complementary information is available indicating that a minimum stimulus intensity is required for a behavioral response to occur, and that the linear form is simpler. These are two very weak arguments. However, even if the details require further investigations, the whole study remains a solid argument in favor of the statement that deposition is significantly stimulated by object perception and that this mechanism is essential in the spatial self-organization of objects. The objective of raising our understanding of individual behavior is therefore reached and the considerable remaining uncertainties associated to the object perception stimulus only reflect the limited knowledge that we presently have of the way an ant perceives its immediate environment and the way it processes the gathered information.

Along the same line of assessing what can or cannot be considered a reliable result, modeling ant movement in [41] has a quite different status that brings us back to the question of introducing effective parameters. The time decorrelation assumption and the instantaneous direction change assumption imply indeed rigorously that any type of detailed two dimensional thigmotactic movement leads to exponentially distributed one dimensional free times the way we defined them. The clockwise and counterclockwise direction change frequency 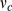 plays therefore the role of an effective parameter in the one dimensional model that can encompass a wide variety of possible ways in which two dimensional movement of ants is affected by the arena border. As a matter of fact, the question of modeling the details of the thigmotactic behavior was therefore not addressed in [41], and this was only possible, without weakening the analysis of the picking up and deposition behaviors, because the role of 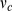 as a meaningful effective movement parameter could be established on a rigorous theoretical basis during the quantitative translation step. This approach lowered significantly the complexity level of the one dimensional object clustering analysis of [41], but it could not be extended, in any straightforward manner, to the analysis of two dimensional clustering or even three dimensional construction behaviors: outside the particular one dimensional case, thigmotactism interacts so closely with picking up and deposition that no convincing conclusion can be reached with regard to the two dimensional morphogenesis mechanisms without increasing our level of understanding of the thigmotactic behavioral details.

Up to this point, we concentrated the discussion on clarifying the exposition of the results of a behavioral modeling attempt, in particular pointing out the parts of the final model that have been established with enough confidence to be considered as useful contributions to cognitive and biological research despite the difficulties intrinsically associated to the collective behavior modeling context. However, the strongest benefit of explicitly distinguishing the above presented methodological steps is certainly elsewhere: it is a strong support and helps avoiding confusion during the process of setting up the model in an interactive manner with experimental design and inversion attempts, including all kinds of back and forth modeling strategies. Parts of the representation of the individual behavior may indeed change status, during the investigation process, according to the success or failure of their experimental validation and characterization procedures. And depending on their status, the argumentative requirement changes significantly. Solid cognitive arguments are required when the considered behavior is chosen to be presented as part of the initial model enunciation, whereas these requirements vanish (and are replaced by only the statistical rigor of the inversion procedures) when the same behavior has the status of an arbitrary choice during the quantitative translation step. A very illustrative example of such back and forth modeling strategies and of their associated methodological requirements is the cockroach aggregation model described in [24]. In the course of this analysis a first experimental protocol led to the measurement of the survival function corresponding to the time cockroaches remain stopped before starting a new movement. It appeared without any ambiguity that the survival was not exponential, which led to a first conclusion that the time decorrelation assumption could not be part of the model enunciation despite of its established validity for several other behaviors with the same species. The memory usage representation was then limited to a simple fit of the survival curve, with the only constraint that the fit be statistically acceptable. This had obviously a quite limited value in terms of cognitive and behavioral interpretation. But later in the investigation it was noted that two kinds of stopping behaviors were at work (a resting stop and a vigilance stop) and that the previous survival function was not exponential because it was the combination of two distinct exponential survival curves. From this observation, the behavioral model enunciation was modified and the temporal decorrelation assumption came back in, together with the detailed definition of the two stopping behaviors. Consequently, the argumentation requirements changed drastically. It was not only required to check experimentally that the two stopping behaviors, when considered separately, had exponentially shaped survival functions. It was required, above all, that the two introduced stops be closely confronted to the behavioral literature, that solid arguments be put forward to justify such a distinction as far as this particular species was concerned, and that the criteria used to identify the stopping type in the experiments (essentially the antenna activity) be accurately defined in accordance with the formulated behavioral arguments. Changing the model enunciation therefore strongly modified the type and amount of efforts required in both the theoretical and experimental fields, with the consequence that the cognitive conclusion of [24] had a much broader significance and could be exposed with a higher level of confidence.

Such very common trial and error modeling practices make it obviously difficult to preserve the rigor required to get a final model with any further significance than the ability to reproduce the observed collective behaviors. From this point of view, a constant reference to a detailed methodological frame is undeniably very helpful. What is known in the relevant behavioral literature? What are the required observations and measurements, considering the cognitive assumptions to be validated? What are the required replication numbers considering the statistical inversions to be performed? The answers to these questions change several times during any detailed investigation, and the above presented methodological framework should help in identifying the most significant of these changes, as consequences of gradual modifications of the model enunciation. There is therefore at least one very practical benefit to be expected: a reduction of the length and complexity of experimental campaigns thanks to a more accurate anticipation of the required biological and statistical argumentations.

We think that such methodological considerations can be of practical help in a broad context of collective behavior studies, but only wherever it is possible to design specific experimental protocols for exploring the pertinence of the suggested individual behavior components ( *specific* meaning that they are independent of the collective scale observations). Object clustering in ants meets such requirements and was used here for the sake of illustrative coherence, but numerous other examples could have played a similar role. Recent studies have indeed been reported that rely implicitly on the approach that is explicitly developped in the present paper. They investigated various collective phenomena, for instance cockroach aggregation [24], fish swarms [66], sheep herds [67] or human crowds [68]–[70].

## Methods

**Figure 7:**
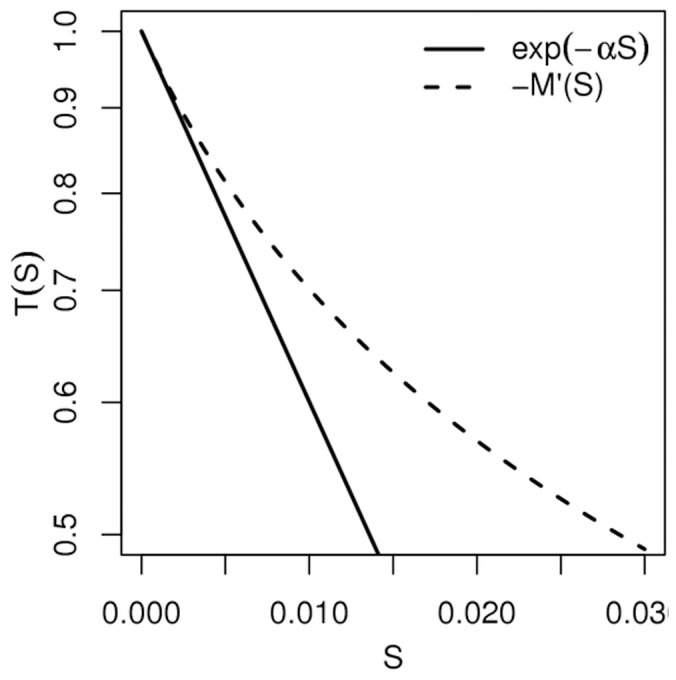
Significance of inter-individual variabilities and temporal correlations. The survival probabilities 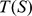 of models 5 or 6 (also to be interpreted as the population average of the survival probabilities of models 3 or 4) compared to an exponential fit corresponding to small 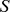 values (short time depositions). The extinction coefficient of the exponential is 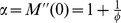.

In the first part of the results section we have mentioned six models leading to identical collective pattern dynamics. In each model, unloaded ants are assumed to be distributed uniformly along the border of the arena and their lineic density is constant in time. As it is further assumed that objects do not overlap, the uniformity of the unloaded ant distribution implies that all objects are encountered with the same rate. The picking up behavior is therefore entirely characterized by the probability 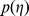 to pick up an encountered object as a function of the perceived density 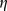 at the object location (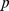 also depends on the activity level 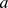, and is noted 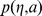, when inter-individual variability is accounted for). After picking up an object, the loaded ant moves in all six models according to a one-dimensional diffusion random walk (characterized by a constant walking speed 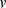 along the arena’s border and a constant rate 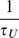 at which U-turns occur). The six models differ by the choices made for each model in terms of inter-individual variability, temporal correlation, and picking up/deposition dependence on perceived object density 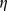. The deposition statistics is entirely characterized by the survival probability 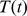 (the probability that the ant carries the object longer than 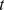) that depends on the perceived density along the path, i.e. on 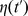 at each time 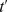 in the 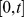 interval (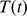 also depends on 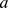, and is denoted 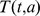, when inter-individual variability is accounted for). Inter-individual variability is characterized by the probability density function of the activity level 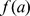. In all the six models, the deposition behavior is stimulated by 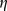. In model 1, the picking up behavior does not depend on 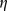, there is no inter-individual variability and no individual temporal correlation: this implies that 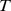 decreases exponentially with time. Model 2 differs from model 1 only by the fact that the picking up behavior is inhibited by 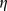. Models 3 and 4 are identical to models 1 and 2, respectively, except that they involve inter-individual variability for the picking up and deposition behaviors: 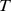 decreases also exponentially with time for each individual, but the decrease rate depends on the activity level 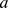. Models 5 and 6 are also identical to models 1 and 2, respectively, except that they involve temporal correlation for the deposition behavior, and therefore 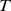 decreases non-exponentially with time. The parameter values and functional dependences for 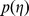 or 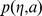, 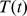 or 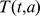, and 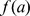 for each model are listed in Tab. 1. Fig. 7 illustrates that the retained parameter values exclude that the survival probability in models 5 and 6 be fitted with any equivalent exponential law. The conclusion is strictly identical when considering the integration over the whole ant population of the survival probability in models 3 and 4. This confirms that temporal correlation is indeed significant in models 5 and 6, and that inter-individual variability is significant in models 3 and 4 (the population average behavior cannot be replaced by that of a uniform population with an equivalent constant activity level).

The theoretical establishment of the indistinguishability of the models with inter-individual variability (models 3 and 4, where 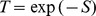) from those with temporal correlations (models 5 and 6, where 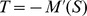) is based on the following mathematical properties of inverse gaussian distributions [71] (see the caption of Tab. 1 for the complete definitions of 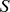, 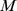 and 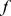): 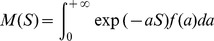 leading to
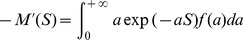
(1)and



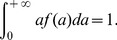
(2)In model 5, when an ant encounters an object it picks it up with a probability 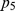 that is independent of 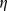 (unlike in Table 1 we temporarily introduce subscripts to make a distinction between models). In model 3, the picking up probability depends on 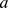 according to 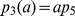. Eq. 2 leads to
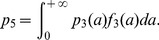
(3)


**Table 1:** Parameter values and functional dependences for the six models of object clustering behavior.

	Functional forms	Parameter values
Model 1 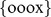	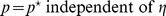	
	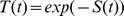	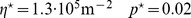
	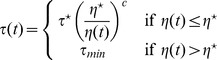	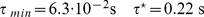 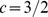
Model 2 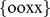 *picking up inhibition*	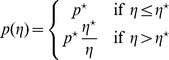	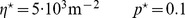
	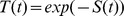	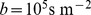
	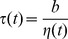	
Model 3 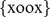 *inter-individual variability*	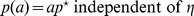	
	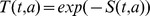	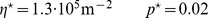 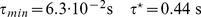
	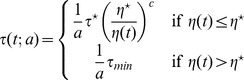	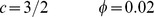
Model 4 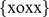 *picking up inhibition & inter-individual variability*	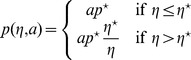	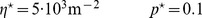
	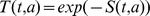 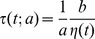	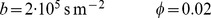
Model 5 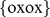 *temporal correlation*	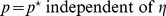	
	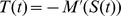	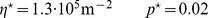
	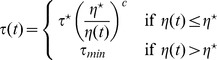	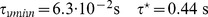 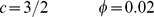
Model 6 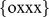 *temporal correlation & inter-individual variability*	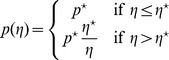	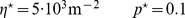
	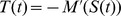	
	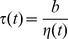	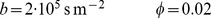

The code in curly brackets indicates, for each model, whether inter-individual variability, temporal correlation, picking up inhibition by 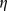 or deposition stimulation by 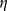 occur (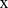) or not (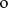). The six models only differ by the functional dependences of the probability 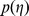 that an ant picks up an encountered object on the perceived density 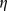, and of the survival probability 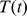 that a loaded ant carries the object longer than time 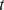 on 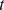 (or 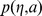 and 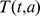, where 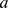 is the ant’s activity level, when inter-individual variability is accounted for). For models 3 and 4, the activity levels are distributed according to an inverse gaussian distribution: 
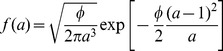
. For models 5 and 6, temporal correlation is significant: 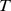 decreases as the opposite of the derivative of a Malkmus transmittivity function 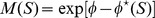 with 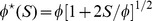, a choice that is inspired by the physics of gaseous radiation [71] where distinct absorption rates at different frequencies lead to non-exponential spectrally integrated extinctions. In all six models, 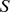 is deduced from the deposition mean free time 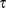 according to 
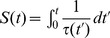
, the walking speed is 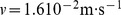 and the U-turn rate is 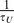 with 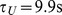.

The population average value of the picking up probability of model 3 equals therefore that of model 5, which implies that each object is picked up with the same temporal statistics in models 3 and 5. When considering models 4 and 6 the only difference is that picking up probabilities depend on 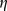 but the proof is the same with
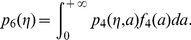
(4)


Very similarly, Eq. 1 leads to
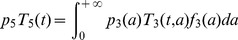
(5)and



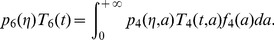
(6)As all ants follow the same statistical moves, the product 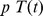 reflects the object’s spatial transition statistics, i.e. the probability density function corresponding to an object being picked up at a given location and deposited at another given one. The derivative with time of 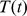 is indeed proportional to the carrying time probability density function and is translated into the distribution of deposition locations via the diffusion random walk statistics. Eqs. 5 and 6 establish therefore that the spatial distribution of deposition locations are strictly identical in models 3 and 5, as well as in models 4 and 6.
